# Genome-Wide Quantification of the Effect of Gene Overexpression on *Escherichia coli* Growth

**DOI:** 10.3390/genes9080414

**Published:** 2018-08-16

**Authors:** Hao Chen, Sumana Venkat, Jessica Wilson, Paige McGuire, Abigail L. Chang, Qinglei Gan, Chenguang Fan

**Affiliations:** 1 Cell and Molecular Biology Program, University of Arkansas, Fayetteville, AR 72701, USA; hc019@uark.edu (H.C.); sv009@uark.edu (S.V.); 2 Department of Chemistry, University of Arkansas, Fort Smith, AR 72913, USA; jwilso18@g.uafs.edu; 3 Department of Biological Sciences, University of Arkansas, Fayetteville, AR 72701, USA; plmcguir@uark.edu; 4 Fayetteville High School, Fayetteville, AR 72701, USA; a.chang@g.fayar.net; 5 Department of Chemistry and Biochemistry, University of Arkansas, Fayetteville, AR 72701, USA; qingleig@uark.edu

**Keywords:** *Escherichia coli*, recombinant protein production, gene overexpression, growth effect, ASKA collection, codon bias, branched-chain amino acids, gene ontology

## Abstract

Recombinant protein production plays an essential role in both biological studies and pharmaceutical production. *Escherichia coli* is one of the most favorable hosts for this purpose. Although a number of strategies for optimizing protein production have been developed, the effect of gene overexpression on host cell growth has been much less studied. Here, we performed high-throughput tests on the *E. coli* a complete set of *E. coli* K-12 ORF archive (ASKA) collection to quantify the effects of overexpressing individual *E. coli* genes on its growth. The results indicated that overexpressing membrane-associated proteins or proteins with high abundances of branched-chain amino acids tended to impair cell growth, the latter of which could be remedied by amino acid supplementation. Through this study, we expect to provide an index for a fast pre-study estimate of host cell growth in order to choose proper rescuing approaches when working with different proteins.

## 1. Introduction

After the whole-genome sequences of thousands of organisms have been well documented, overexpressing genes to get highly pure proteins for further characterization and engineering becomes an indispensable part of biochemistry, molecular biology, cell biology, and synthetic biology. Moreover, among the 239 US-FDA (Food and Drug Administration) approved therapeutic peptides and proteins, as well as their 380 drug variants, the majority are manufactured by recombinant protein production [1]. 

In both basic research and drug production, *Escherichia coli* is one of the most widely-used hosts to express recombinant proteins due to a number of advantages. First, it grows quickly, with a doubling time of about 20 min in rich growth media [2], which means the total time of expressing target proteins, from inoculation to cell harvest, is only a few hours in most circumstances. Second, it readily reaches a high cell density for good protein yields. Commonly, 1 to 2 g dry cell weight or 10^13^ cells could be obtained from 1 L of liquid Lysogeny broth (LB) medium [2]. Third, it is cheap and easy to make growth media for *E. coli* such as the LB medium and the Terrific Broth (TB) medium. Fourth, the genetics of *E. coli* is well known, and it is convenient to remove certain genes from the genome for different purposes [3]. Fifth, it is easy to introduce heterologous genes into *E. coli* by plasmid transformation. Last but not least, a large number of vectors, fusion tags, and mutant strains have been developed for optimal expression of target proteins in *E. coli*. Several review articles have been published recently to cover these topics [4,5].

A commonly encountered problem for recombinant protein production is impeded cell growth or reduced biomass accumulation. There are two major reasons for this phenomenon. The first is the general metabolic burden, which could be explained as the competition between biomass accumulation and recombinant protein production for metabolic materials such as cellular energy, ATP, and substrates, amino acids, [6]. This competition leads to stress responses including the stringent response and RNA polymerase subunit S-mediated stress responses, which could further decrease or even inhibit cell growth [7]. This competition also causes increased protease activities for the overexpressed proteins [8]. The second reason is the specific protein toxicity, which is caused by the harmful functions of overexpressed proteins on normal proliferation and homeostasis of host cells [9]. For the metabolic burden, several improving approaches have been developed such as decreasing inducer concentrations, lowering plasmid copy numbers, and adding more nutrients in growth media [10,11]. Although the protein toxicity could depend on individual proteins case by case, we aimed to find general features to facilitate recombinant protein production.

For this purpose, we utilized the a complete set of *E. coli* K-12 ORF archive (ASKA) collection, which is a complete set of *E. coli* strains for overexpressing individual *E. coli* K-12 genes [12]. Although the authors who constructed the ASKA collection also tested the cell growth of individual strains in the library qualitatively by using the LB agar plate, the determination of growth effects was not clearly described, and the list of genes which impaired cell growth was not provided [12]. Thus, in this study, we quantified the effects of overexpressing individual *E. coli* genes on its own cell growth and combined the results with bioinformatical analyses to identify shared features of proteins, which could hamper cell growth. 

## 2. Materials and Methods

### 2.1. Strain and Plasmid Construction

The ASKA (−) collection was obtained originally from the Coli Genetic Stock Center at Yale University. The no insert control of pCA24N was constructed by the Q5 Site-Directed Mutagenesis Kit (New England BioLabs, Ipswich, MA USA), with the F primer: 5′-taagggtcgacctgcagccaagc-3′ and the R primer: 5′-atccgtatggtgatggtgatggtgagatcc-3′. The plasmid pCA24N-*gfp* was constructed by the HiFi DNA Assembly Cloning Kit (New England BioLabs) with the F primer: 5′-gaattcattaaagaggagaaattaactatgagcaagggcgaagaactgtttacgg-3′ and the R primer: 5′-ctaattaagcttggctgcaggtcgacccttaatgatgatgatgatgatgtgagcctttatacag-3′. The gene of green fluorescent protein (GFP) was expressed under the control of the same promoter used for the ASKA strains. The *E. coli* AG1 strain, which is the host strain of the ASKA collection was purchased from Agilent Technologies (Wilmington, DE, USA).

### 2.2. Cell Growth Experiments

Individual plates of the ASKA collection were replicated by inoculating 3 µL stock culture into 150 µL fresh LB media with 50 µg/mL chloramphenicol in each well of 96-well plates, and incubated at 37 °C overnight. The absorbance at 600 nm of each well was then read by the microplate reader. The overnight culture in each well was diluted to OD_600nm_ = 0.15 with a total volume 150 µL of fresh LB media with 50 µg/mL chloramphenicol. Each plate had three biological replicates. The 96-well plates were sealed with oxygen-permeable membranes (Sigma-Aldrich, St. Louis, MO, USA). The cell growth was monitored by reading the absorbance at 600 nm with microplate readers at 37 °C continuously. The doubling time was calculated by the equation: Doubling time = lg2/lgX. X is the growth rate in the exponential phase, which was automatically provided by Gen5 software designed for the BioTek microplate reader (Winooski, VT, USA). The monitoring of GFP expression by fluorescence followed previous studies [13,14].

### 2.3. Bioinformatical Analyses

The software and online resources used for bioinformatical analyses were described in each subsection of Results and Discussion.

## 3. Results and Discussion

### 3.1. Growth Condition Selection

**Figure 1:**
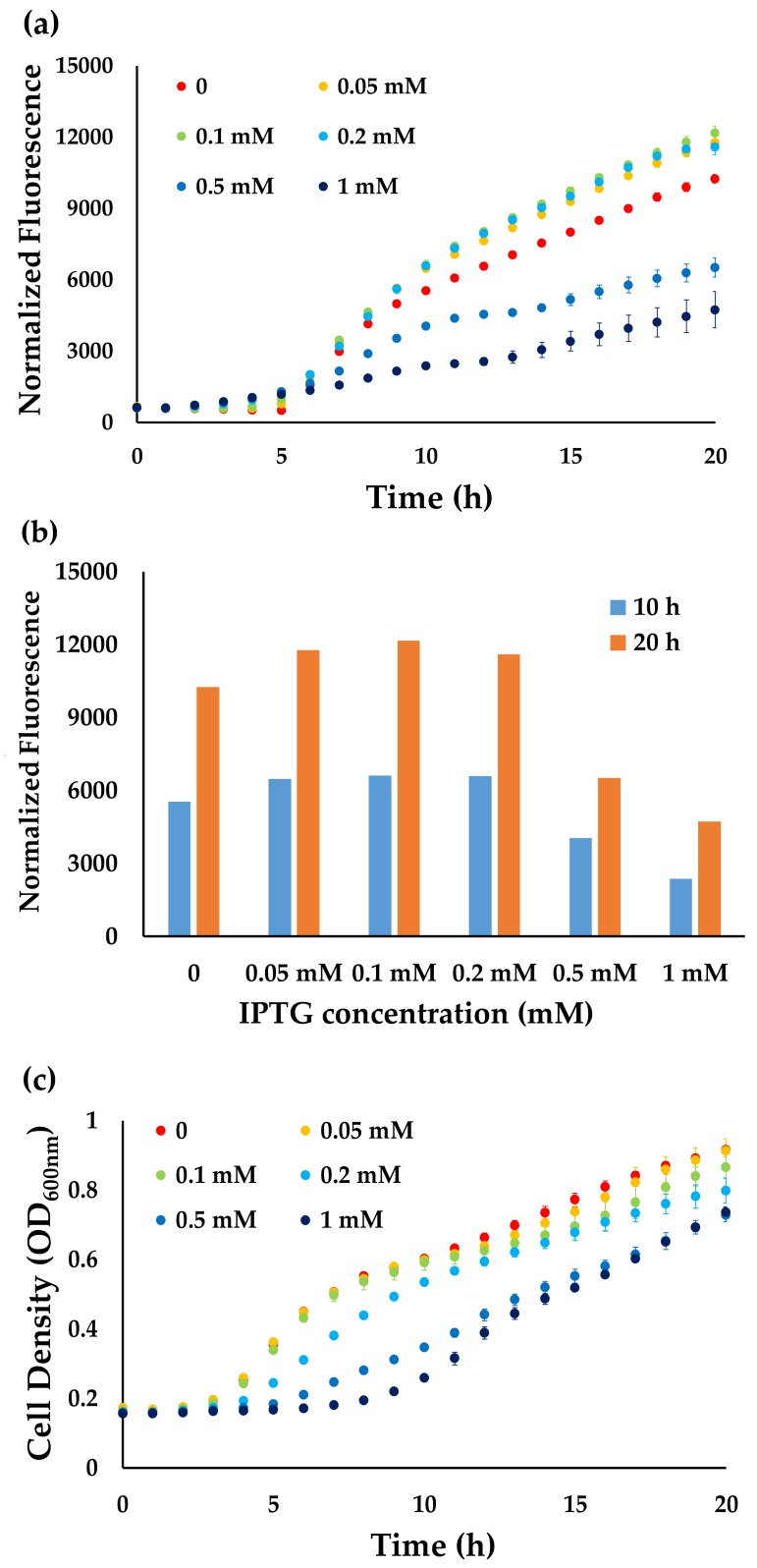
The effects of isopropyl β-d-1-thiogalactopyranoside (IPTG) concentrations on green fluorescent protein (GFP) expression and cell growth. (**a**) The normalized fluorescence intensity was used for determining protein production, which was the absolute fluorescent intensity subtracted with the fluorescence background of AG1 cells in the same growth condition over the cell density (OD_600nm_); (**b**) The effects of IPTG concentrations on the normalized fluorescence intensity at 10 h and 20 h after inoculation; (**c**) The cell density was used for determining cell growth. The mean values and standard deviations were calculated from three biological replicates.

First, GFP was used as a reporter to determine the optimal concentration of the inducer isopropyl β-d-1-thiogalactopyranoside (IPTG) for high-throughput growth tests. We monitored both recombinant protein production by the fluorescence intensity (Figure 1a,b) and biomass accumulation by OD_600nm_ (Figure 1c). Interestingly, there was a high fluorescence reading, even without IPTG in the growth medium, indicating that the pCA24N vector is not tightly controlled. Lower concentrations of IPTG (0.05 to 0.2 mM) significantly increased the GFP expression (*p* < 0.01 by the *t*-test), while commonly used concentrations of IPTG (0.5 to 1 mM) decreased the GFP expression significantly (Figure 1a). This result was consistent with previous studies, which showed that decreasing concentrations of inducers could enhance recombinant protein production [6]. On the other hand, the concentration of IPTG also affected cell growth. Starting from 0.2 mM, higher concentrations of IPTG hindered normal cell growth (Figure 1b). Considering both recombinant protein production and biomass accumulation, 0.05 mM IPTG was chosen for high-throughput growth tests, since this concentration provided the best protein yield without negative effects on cell growth.

### 3.2. High-Throughput Growth Tests of the ASKA Collection

With 0.05 mM IPTG as the inducer, the doubling time of the strain containing the no insert control of the pCA24N vector was 42 min, which is longer than the previously reported 20 min [2]. This was because that 96-well plates used in this study have relatively smaller top space and lower oxygen supply than regular culture tubes. To better demonstrate the growth effect of overexpressing individual genes on cell growth, delay factor was defined as the ratio of the doubling time of individual strains in the ASKA collection over the doubling time of the strain harboring the no insert control of the pCA24N vector. The delay factors for all the 4071 *E. coli* genes tested in this study were summarized in Table 1 and listed in Table S1, respectively. Among them, 921 strains had no or moderate growth effects (delay factor < 2), 3049 strains had significant growth effects (delay factor between 2 and 7), and 101 strains had severe growth effects (delay factor > 7). More than 75% of strains had significant or severe growth effects, indicating that the metabolic burden could be a general issue in recombinant protein production. Only a small portion of strains severely impaired cell growth, possibly due to both the metabolic burden and specific protein toxicity.

### 3.3. Bioinformatical Analyses of Factors Affecting Cell Growth

#### 3.3.1. The Effect of Protein Length on Cell Growth

**Figure 2:**
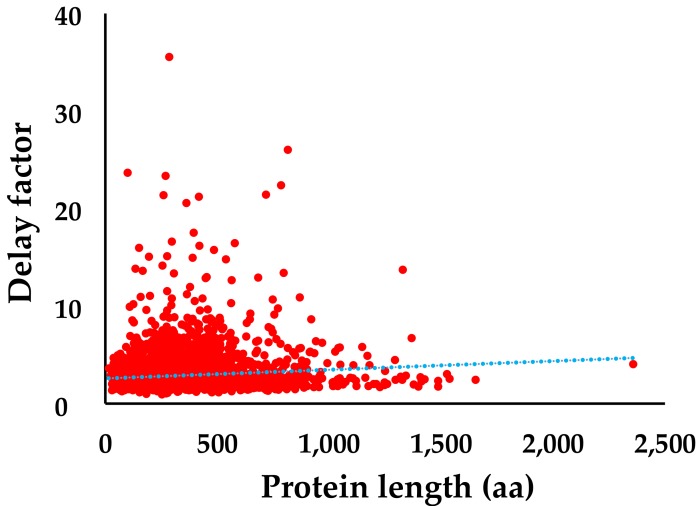
The effects of protein length on cell growth. The delay factor and protein length of individual ASKA collection strains were plotted with red color. The blue dot line is the trend line.

We expected that a longer gene length or protein length needs more materials, thus affecting cell growth. To test this factor, the delay factor versus the protein length of each ASKA collection strains was plotted (Figure 2). The median *E. coli* protein length is 280 aa. The median delay factor for *E. coli* proteins less than 280 aa is 2.37, while that for *E. coli* proteins more than 280 aa is 2.44. The difference is not significant, consistent with the trend line which shows only a slight rise of the delay factor with increasing protein length (Figure 2).

#### 3.3.2. The Effect of Amino Acid Compositions on Cell Growth

**Figure 3:**
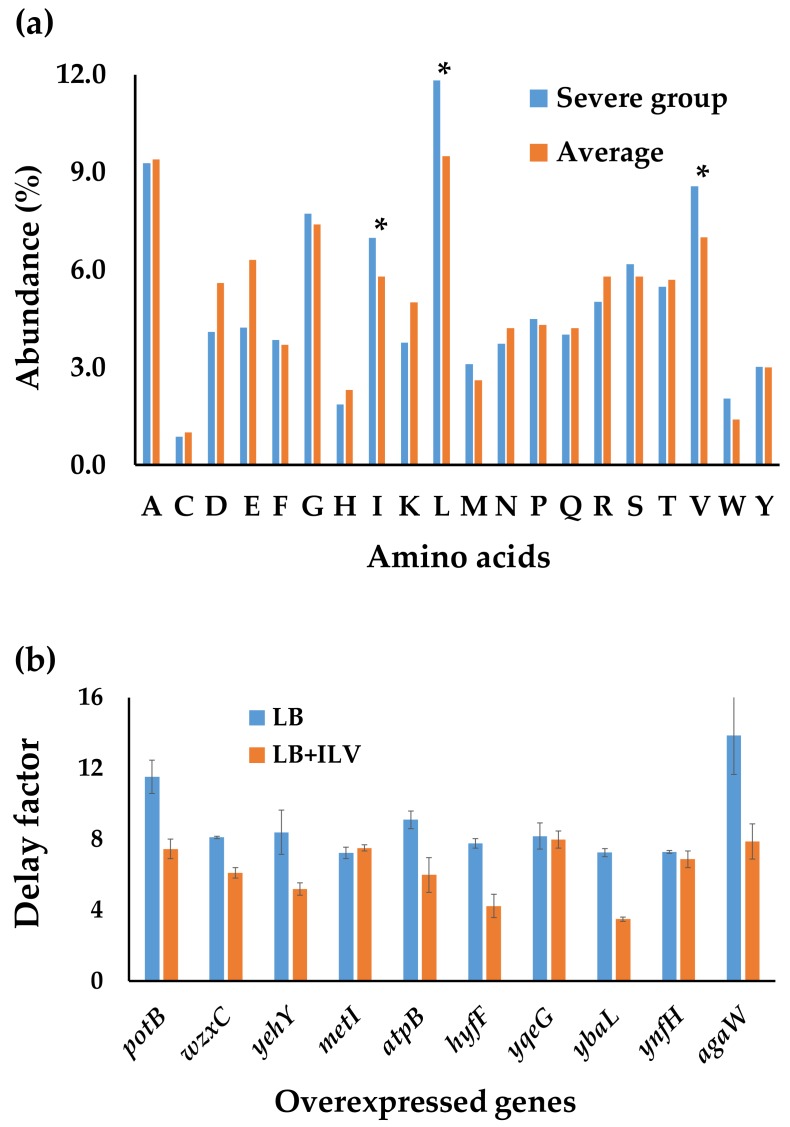
The effects of amino acid compositions on cell growth. (**a**) The abundance of each amino acids in proteins with severe growth effects. One-letter abbreviations of amino acids were used. The *t*-test was used and significant differences (*p* < 0.05) were marked with *. (**b**) The delay factors of selected strains grown in media with or without supplementary branched-chain amino acids (BCAAs). The abundances of ILV in the selected genes are *potB* (37.46%), *wzxC* (36.19%), *yehY* (35.58%), *metI* (35.02%), *atpB* (34.44%), *hyfF* (33.64%), *yqeG* (33.25%), *ybaL* (33.16%), *ynfH* (33.10%), and *agaW* (33.08%). The delay factor was defined as the ratio of the doubling time of individual strains in the ASKA collection over the doubling time of the strain harboring the no insert control of the pCA24N vector. ILV means isoleucine, leucine, and valine. The mean values and deviations were calculated from three biological replicates.

Because the amount of individual free amino acids in cells are different, we assumed that the abundance of each amino acids in a target protein might affect cell growth when overexpressing it. Thus, the amino acid compositions of proteins overexpressed in the strains that had severe growth effects were calculated (Table S2). Comparing with the mean values of all the *E. coli* proteins, the abundances of isoleucine (Ile), leucine (Leu), and valine (Val) are significantly increased in proteins which had severe effects on cell growth (Figure 3a). Interestingly, these three amino acids all belong to branched-chain amino acids (BCAAs), which have been shown to be essential for bacterial growth [15,16]. 

To test if the severe growth effect was really caused by insufficient intracellular BCAAs, we randomly selected ten strains from the severe growth group, which overexpress proteins with high abundance of BCAAs, and tested their growth in LB media supplemented with 2 mM (each) of Ile, Leu, and Val. Most of the strains had improved growth, indicating that overexpressing proteins with high abundance of BCAAs could indeed impair cell growth, which could be then remedied by adding those BCAAs in growth media. (Figure 3b). We also tested the effect of BCAA supplementation on growth of strains expressing proteins with average abundance of BCAAs. The results showed that the improvement was not as significant as that for proteins with high abundance BCAAs (Figure S1).

#### 3.3.3. The Effect of Codon Bias on Cell Growth

**Figure 4:**
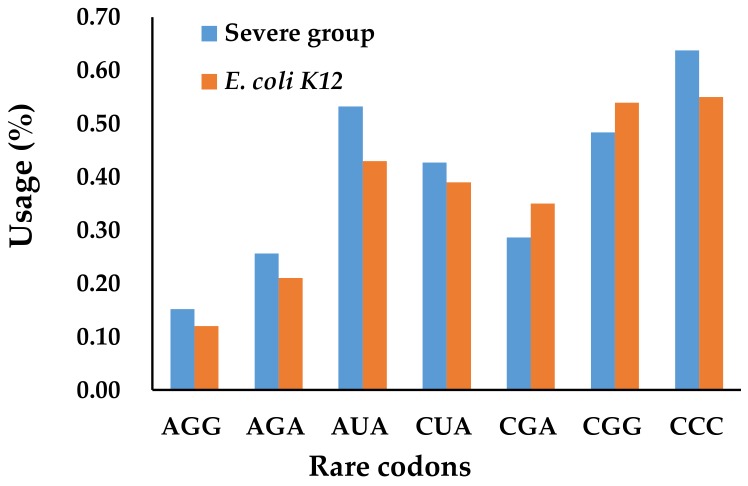
The effects of rare codon usage on cell growth. The codon usage of all the *E. coli* K12 genes was cited from Kazusa DNA Research Institute, Japan (http://www.kazusa.or.jp). The *t*-test was used.

Another common issue in recombinant protein production is codon bias, which means the occurrence of synonymous codons in target genes is largely different from that of host cells. The depletion of rare tRNAs by overexpressing recombinant proteins could cause early termination or mistranslation of recombinant proteins [17,18]. We expected that the shortage of rare tRNAs could also cause similar problems for native protein production, thus affecting cell growth. To test this assumption, the rare codon usage in all the *E. coli* K12 proteins and in the group with severe growth effects was compared (Figure 4, listed in Table S3). We focused on the seven rare codons in *E. coli* K12 cells, which are AGG, AGA, CGA, and CGG for arginine, AUA for isoleucine, CUA for leucine, and CCC for proline [19]. Unexpectedly, no significant differences were observed between the severe group and all the *E. coli* K12 proteins in rare codon usage.

The result is consistent with previous studies, which have shown that tRNA availability for rare codons is not the most important factor for protein production during gene overexpression [20,21]. Common strategies for dealing with codon bias include codon optimization and special strains harboring rare tRNAs in plasmids [22]. However, these approaches were reported to cause mRNA instability and protein aggregation [23,24], as rare codons could play important roles in forming specific RNA secondary structures for its stability and interaction with ribosomes [20,25,26], and in translational pausing which could help proper protein folding [27].

#### 3.3.4. Gene Ontology Analyses

**Figure 5:**
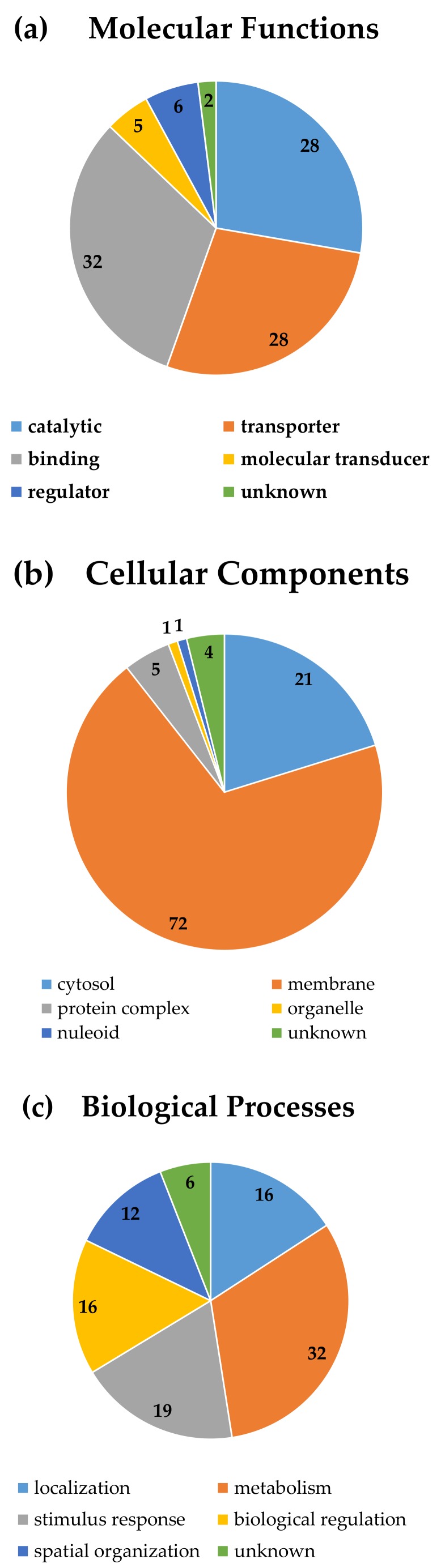
Gene ontology of proteins overexpressed in strains with severe growth effects. (**a**) Molecular functions; (**b**) Cellular components; and (**c**) Biological processes.

In the above three subsections, we focused on the metabolic burden resulting from the general properties of proteins rather than their functions. From this subsection, we started to consider the protein toxicity associated with their functions. We first categorized proteins which were overexpressed in strains with severe growth effects into different groups according to their annotated molecular functions, cellular components, and biological processes in UniProt-GOA database [28] (Figure 5).

For molecular functions, those proteins are distributed evenly in the three major categories: Enzymes, transporters, and binding proteins. Compared with the analysis of all the *E. coli* genes (Figure S2a), the fraction in transporters was significantly higher in the group with severe effects (*p* < 0.01 by the *t*-test). For cellular localization, most of them are associated with membranes, which is consistent with the previous analysis of amino acid compositions, since membrane proteins tend to have higher abundances of nonpolar amino acids, including BCAAs due to their interactions with membrane lipids. Compared with the analysis of all the *E. coli* genes (Figure S2b), the fraction in membranes was significantly higher in the group with severe effects (*p* < 0.01 by the *t*-test). For biological processes, they span on all essential cellular processes. Compared with the analysis of all the *E. coli* genes (Figure S2c), the fraction in metabolism was significantly lower in the group with severe effects (*p* < 0.01 by the *t*-test).

#### 3.3.5. Protein Functional Interaction Network Analyses

**Figure 6:**
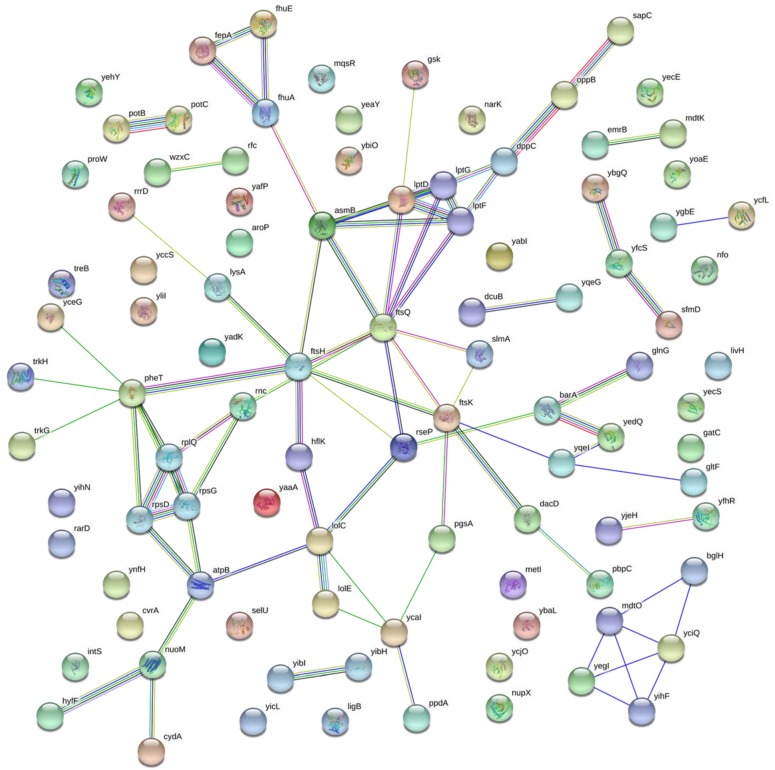
The functional interaction network analysis of proteins with severe growth effects by using STRING database. Network nodes represent proteins, and splice isoforms or post-translational modifications are collapsed. Edges represent protein-protein associations, and associations are meant to be specific and meaningful.

Next, we analyzed the functional interaction network of proteins in the severe growth group by STRING database (http://string-db.org) [29] (Figure 6). Consistent with the gene ontology analyses, which demonstrated that target proteins are distributed evenly in different functional categories and biological processes, the interaction map only showed three clusters of proteins with five–six members.

One cluster includes *asmB*, *lptD*, *lptG*, *dppC*, *lptF*, and *ftsQ*. LptD, LptF, and LptG are three essential proteins in the lipopolysaccharide transport system [29,30,31]. DppC is a membrane subunit for dipeptide ABC transporter [32]. FtsO is an essential cell division protein, which is required for localization of transporter proteins to the cell poles [33]. AsmB is also associated with cell division and involved in lipid A biosynthesis. Clearly, overexpression of these genes could interfere with normal cell division and membrane formation, which are essential for cell growth.

Another cluster contains *pheT*, *rplQ*, *rpsD*, *rpsG*, and *rnc*. RplO, RpsD, and RpsG are components of ribosomes [34,35,36]. RNase III (Rnc) is a key enzyme in rRNA processing [37]. PheT is one subunit of phenylalanyl-tRNA synthetase, which has the binding site with tRNA^Phe^ and editing activity [38]. Overexpressing these genes may affect the proper assembly of ribosomes and translation fidelity, thus impeding protein biosynthesis and cell growth. Actually, growth effects have also been observed in our studies with other aminoacyl-tRNA synthetases [39].

The last cluster includes *bglH*, *mdtO*, *yciQ*, *yegI*, and *yihF*. BglH is a carbohydrate-specific outer membrane porin [40]. MdtO is a component of a putative multidrug efflux pump [41]. YciQ is involved in membrane integration [42]. YihF and YegI have unknown functions, but they have high gene co-occurrences across genomes with both MdtO and YciO [43]. Again, overexpressing membrane-associated proteins tends to have growth effects, which is consistent with previous plate tests [12].

## 4. Conclusions

In summary, we quantified the effect of overexpressing individual *E. coli* genes on its cell growth. Overexpression of membrane-associated proteins, or proteins with high BCAA abundances, tended to hinder cell growth. For recombinant protein production, it is suggested that the first thing is to check BCAA abundances, and supplementing BCAAs in growth media could recover cell growth when overexpressing proteins with high BCAA abundances. For membrane-associated proteins or proteins related to protein biosynthesis, it is recommended to reduce the rate of protein production with lower inducer concentrations, weaker promoters, or lower copy numbers of vector to improve cell growth for an increased total protein yields. 

## Figures and Tables

**Table 1:** The summary of delay factors of individual a complete set of *E. coli* K-12 ORF archive (ASKA) strains.

Growth Effects ^1^	Delay Factors ^2^	Numbers of Strains	Percentage
No or moderate	<2	921	22.6%
Significant	2 to 7	3049	74.9%
Severe	>7	101	2.5%

^1^ The classification of growth effects was based on delay factors subjectively. ^2^ Delay factors were calculated by dividing the doubling time of individual strains in the ASKA collection by the doubling time of the strain harboring the no insert control of the pCA24N vector.

## Supplementary Materials

The following are available online at http://www.mdpi.com/2073-4425/9/8/414/s1, **Figure S1.** The delay factors of selected strains with average BCAA abundance. **Figure S2.** Gene ontology of all E. coli K12 genes. **Table S1**. The complete list of delay factors for the ASKA collection. **Table S2**. The list of amino acid compositions of proteins overexpressed in strains with severe growth effects. **Table S3**. The list of rare codon abundances in proteins overexpressed in strains with severe growth effects.
