# Supplementary figures and images for: Genome-Wide Quantification of the Effect of Gene Overexpression on Escherichia coli Growth
Source: Genes (Basel). 2018 Aug 16;9(8):414. doi: 10.3390/genes9080414 (PMC6116040; doi:10.3390/genes9080414)

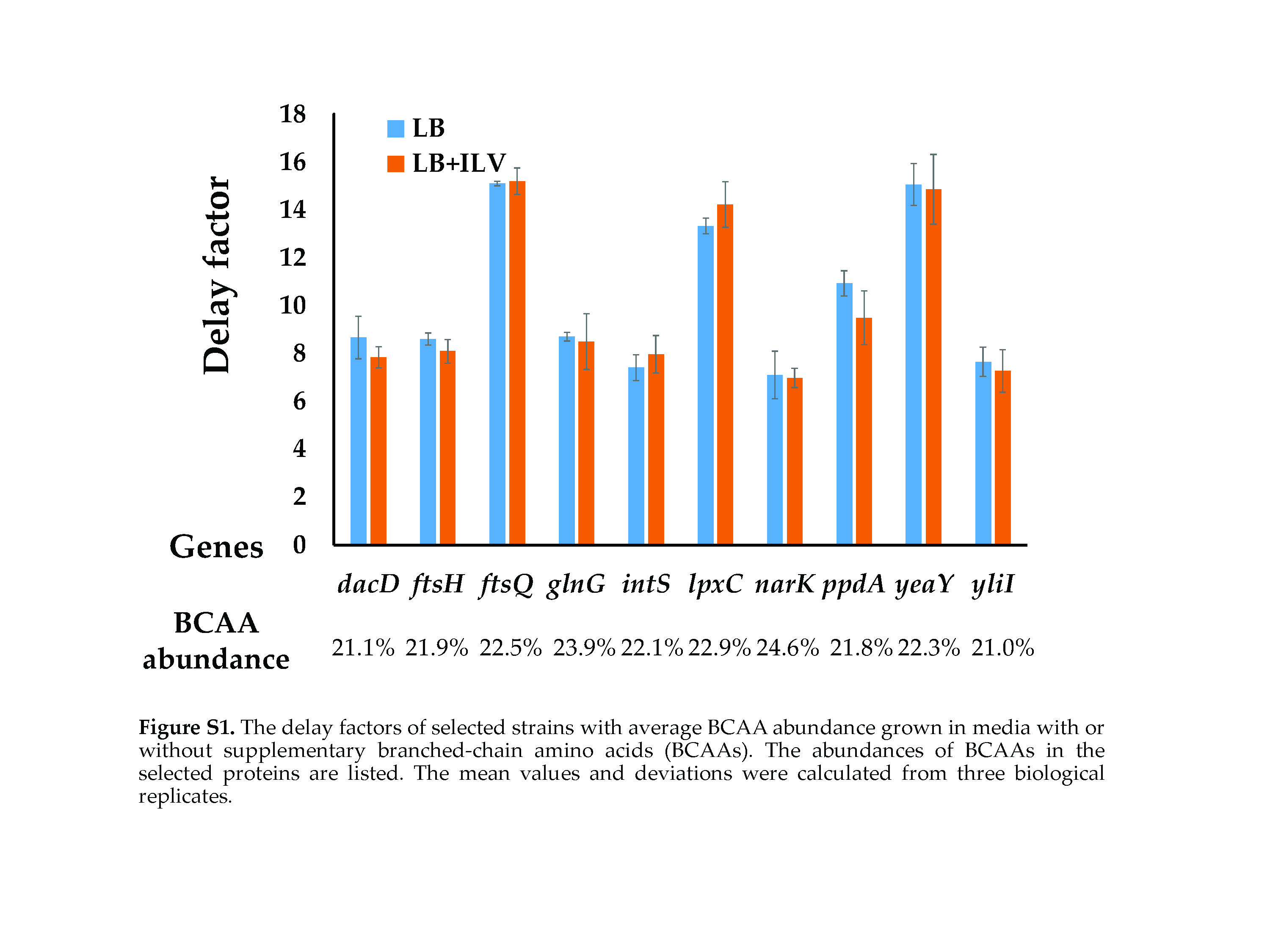

Supplement: Supplementary file 1 [file genes-09-00414-s001.zip › genes-338431 - Supplementary - after proofs_v2/Figure S1.jpg]

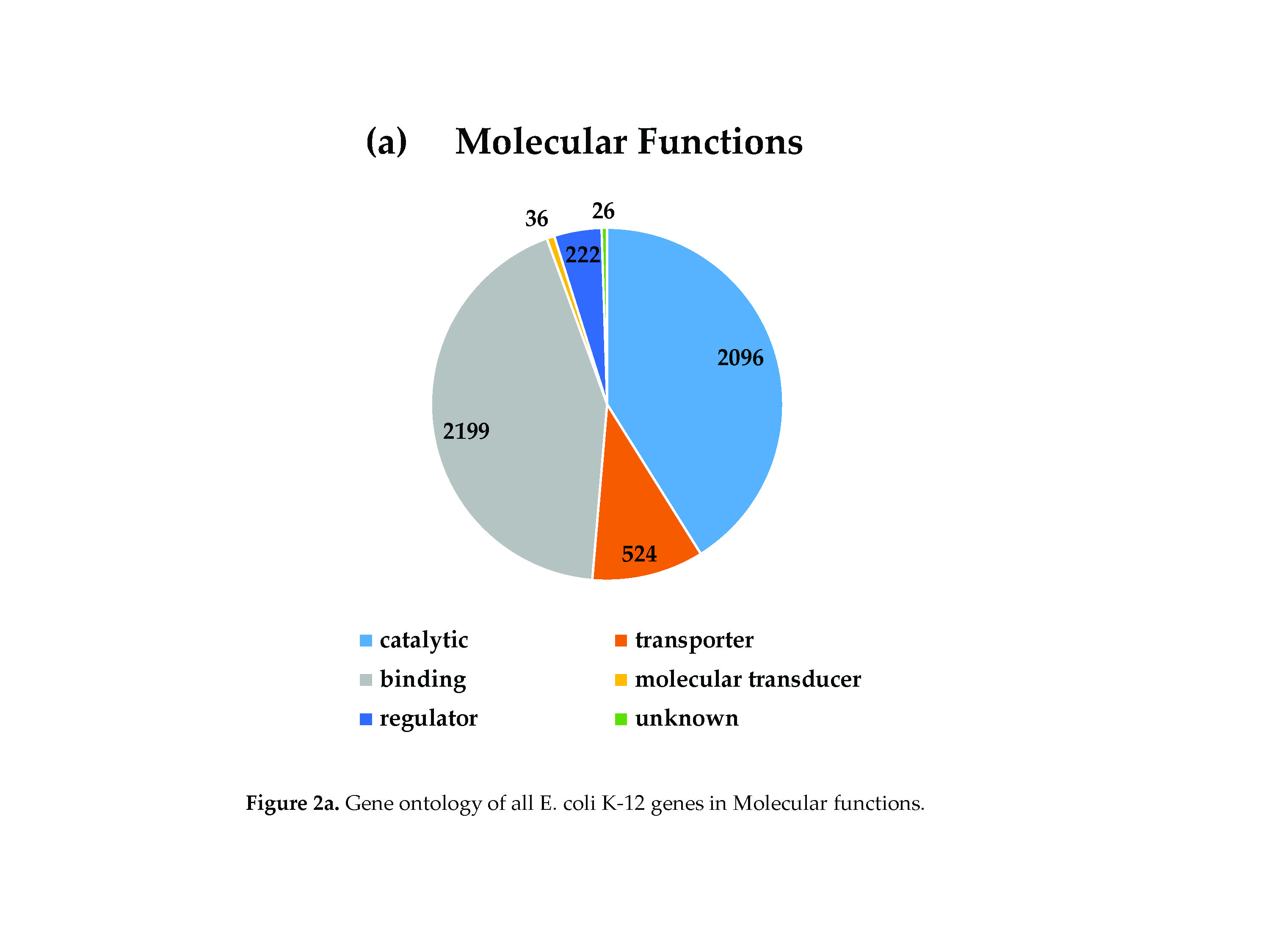

Supplement: Supplementary file 1 [file genes-09-00414-s001.zip › genes-338431 - Supplementary - after proofs_v2/Figure S2a.jpg]

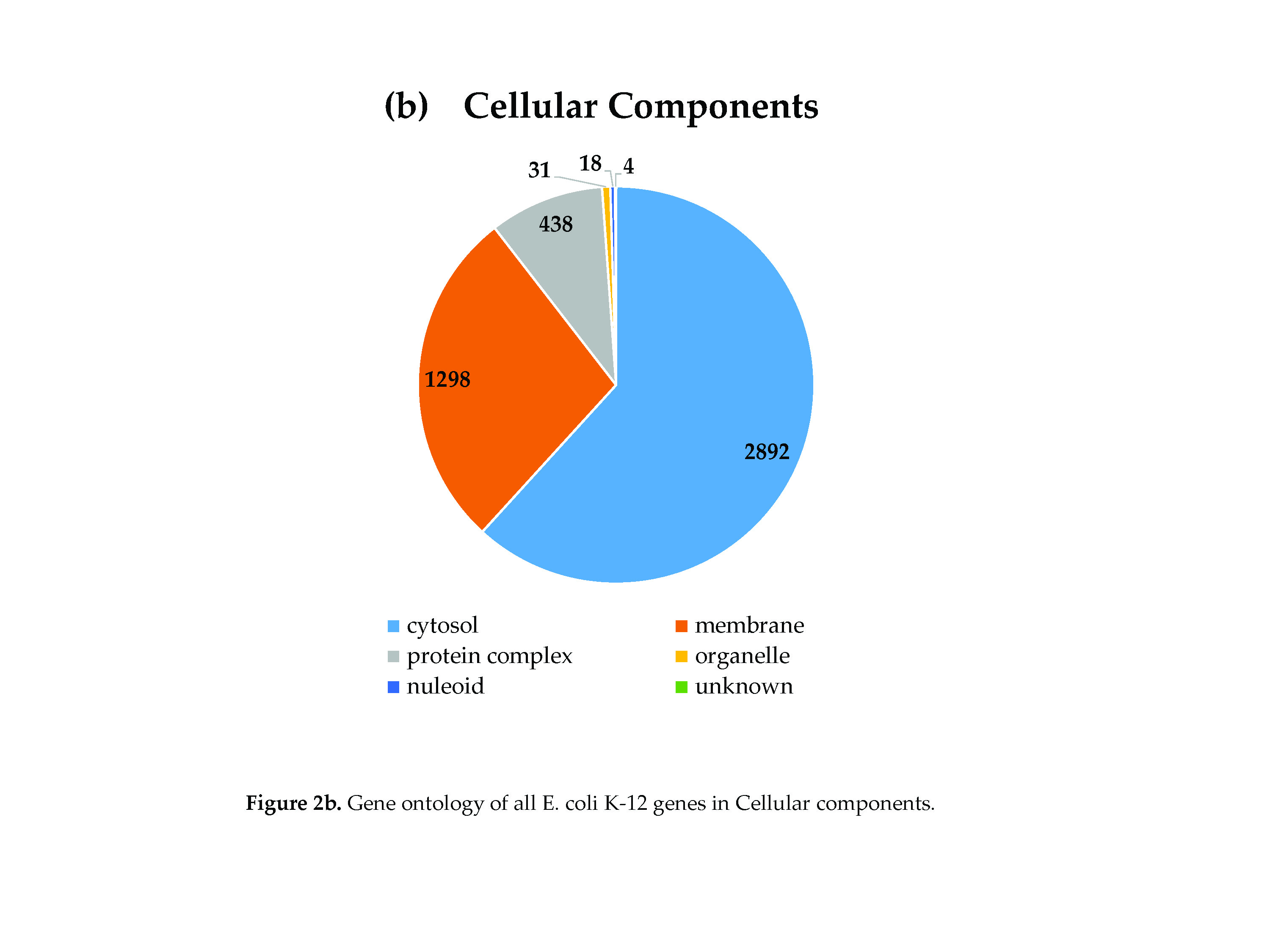

Supplement: Supplementary file 1 [file genes-09-00414-s001.zip › genes-338431 - Supplementary - after proofs_v2/Figure S2b.jpg]

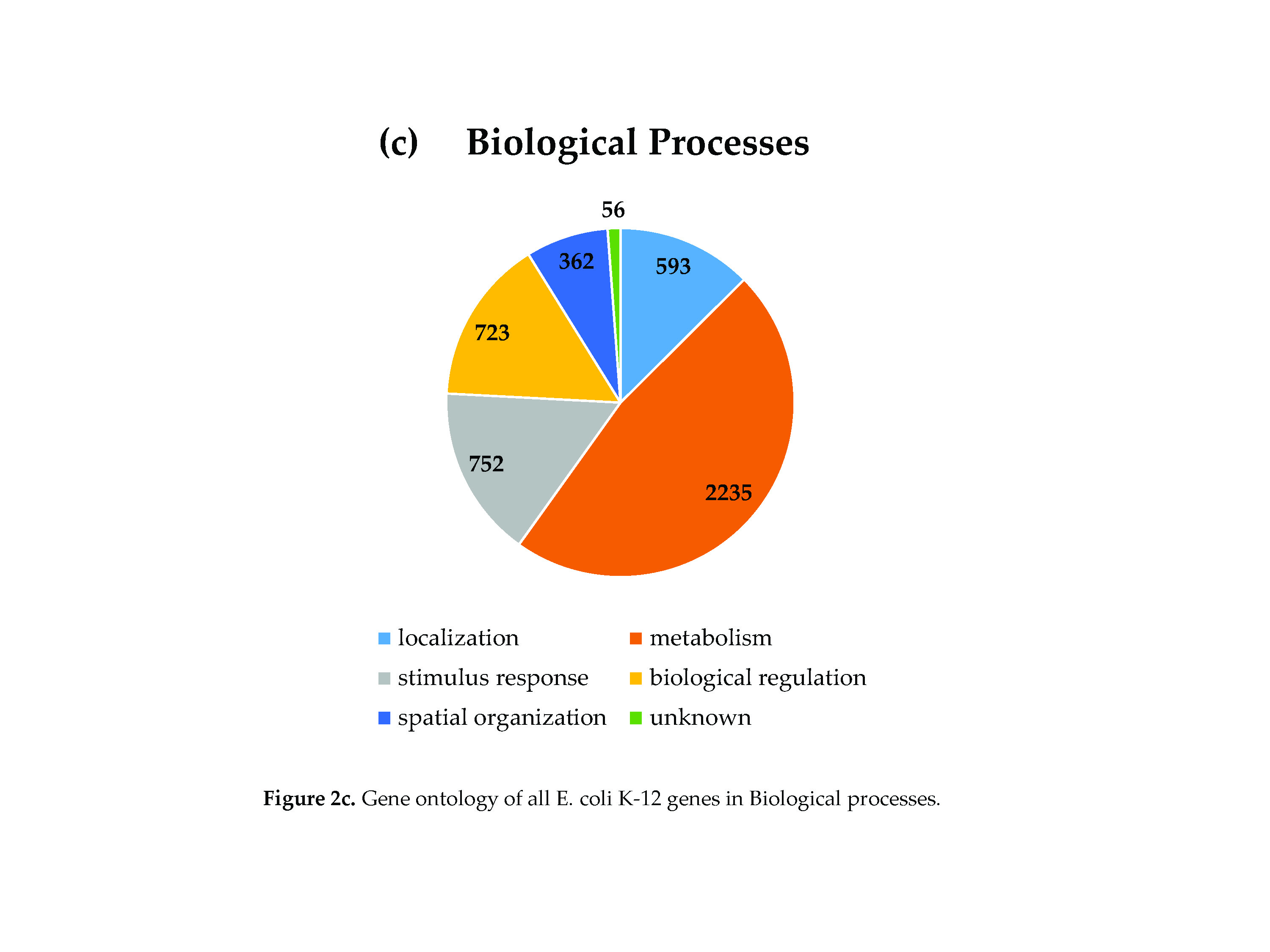

Supplement: Supplementary file 1 [file genes-09-00414-s001.zip › genes-338431 - Supplementary - after proofs_v2/Figure S2c.jpg]
